# First-Line A Direct Aspiration First-Pass Technique vs. First-Line Stent Retriever for Acute Ischemic Stroke Therapy: A Meta-Analysis

**DOI:** 10.3389/fneur.2018.00801

**Published:** 2018-09-25

**Authors:** Kevin Li-Chun Hsieh, Kai-I Chuang, Hsu-Huei Weng, Sho-Jen Cheng, Yu Chiang, Cheng-Yu Chen

**Affiliations:** ^1^Department of Medical Imaging, Taipei Medical University Hospital, Taipei, Taiwan; ^2^Research Center of Translational Imaging, College of Medicine, Taipei Medical University, Taipei, Taiwan; ^3^Department of Radiology, School of Medicine, College of Medicine, Taipei Medical University, Taipei, Taiwan; ^4^Department of Diagnostic Radiology, Chang Gung Memorial Hospital, Chang Gung University College of Medicine, Chiayi, Taiwan; ^5^Department of Respiratory Care, Chang Gung University of Science and Technology, Chiayi, Taiwan; ^6^Department of Psychology, National Chung Cheng University, Chiayi, Taiwan; ^7^Department of Imaging Physics, Division of Diagnostic Imaging, University of Texas MD Anderson Cancer Center, Houston, TX, United States

**Keywords:** ADAPT, stent-retriever, intra-arterial thrombectomy, stroke, thrombosuction, penumbra

## Abstract

**Background:** Recent trials have proved the efficacy of mechanical thrombectomy over medical treatment for patients with acute ischemic stroke, with the balance of equivalent rates of adverse events. Stent retrievers were applied predominantly in most trials; however, the role of other thrombectomy devices has not been well validated. A direct aspiration first-pass technique (ADAPT) is proposed to be a faster thrombectomy technique than the stent retriever technique. This meta-analysis investigated and compared the efficacy and adverse events of first-line ADAPT with those of first-line stent retrievers in patients with acute ischemic stroke.

**Methods:** A structured search was conducted comprehensively. A total of 1623 papers were found, and 4 articles were included in our meta-analysis. The Critical Appraisal Skills Programme tools were applied to evaluate the quality of studies. The primary outcome was defined as the proportion of patients with the Thrombolysis in Cerebral Ischemia (TICI) scale of 2b/3 at the end of all procedures. Secondary outcomes were the proportion of patients with functional independence (modified Rankin scale of 0–2) at the third month, the proportion of patients with the Thrombolysis in Cerebral Ischemia (TICI) scale of 2b/3 by primary chosen device, and the proportion of patients who received rescue therapies. Safety outcomes were the symptomatic intracranial hemorrhage (sICH) rate and the mortality rate within 3 months.

**Results:** One randomized controlled trial, one prospective cohort study, and two retrospective cohort studies were included. No significant difference between these 2 strategies of management were observed in the primary outcome (TICI scale at the end of all procedures, odds ratio [OR] = 0.78), two secondary outcomes (functional independence at the third month, OR = 1.16; TICI scale by primary chosen device, OR = 1.25), and all safety outcomes (sICH rate, OR = 1.56; mortality rate, OR = 0.91). The proportion of patients who received rescue therapies was higher in the first-line ADAPT group (OR = 0.64).

**Conclusions:** Among first-line thrombectomy devices for patients with ischemic stroke, ADAPT with the latest thrombosuction system was as efficient and safe as stent retrievers.

## Introduction

Recent trials and meta-analyses have proved the efficacy of mechanical thrombectomy over medical treatment for patients with acute ischemic stroke, with the balance of equivalent rates of adverse events (1–7). In most of these trials, stent retrievers, such as the Solitaire (Covidien, Plymouth, MN) and Trevo (Stryker, Kalamazoo, MI), have been used. However, the role of aspiration thrombectomy devices has not been well validated.

A direct aspiration first-pass technique (ADAPT) was introduced in July 2013 (8) and has been proposed as a faster thrombectomy technique than the stent retriever technique (9–12). Because better clinical outcomes have been correlated with early vessel recanalization (13), an initial attempt at recanalization by using ADAPT may be warranted with an improvement in the time required for the procedure. Several observational studies have reported comparable rates of recanalization and 90-day functional independence in patients treated with ADAPT and stent retrievers (14–16). It was also proposed that ADAPT technique is a cheaper technique than the stent retriever thus it should be recommended (10). However, the latest Contact Aspiration vs Stent Retriever for Successful Revascularization (ASTER) trial demonstrated that first-line ADAPT did not result in an increased successful revascularization rate at the end of the procedure (17). The rate of the introduction of rescue therapy was also higher in the ADAPT group than in the stent retriever group. Therefore, the purpose of this meta-analysis is to investigate and compare the efficacy and adverse events of first-line ADAPT with those of first-line stent retrievers in patients with acute ischemic stroke.

## Methods

### Article search strategy

A structured search on PubMed, Web of Science, and LISTA (EBSCO) was conducted using the keywords *intra-arterial thrombectomy, thrombosuction, stent retriever, stent-retriever*, and *acute ischemic stroke* through August 2017. We identified all studies that related to comparisons of stent retrievers and ADAPT in patients with acute ischemic stroke. This study was conducted and the results were reported according to the Preferred Reporting Items for Systematic Reviews and Meta-Analyses guidelines (18) (Supplementary Material).

### Article selection

A total of 1623 papers were found. The titles and abstracts of all articles were screened by a first investigator (KH). The second and third investigators (KC and CC) reached a consensus when disagreement occurred. After excluding duplicates, articles that did not focus on our target thrombectomy devices, nonhuman studies, and articles without an available full text, 12 papers were recorded. The 5 studies that included the conventional thrombosuction catheter (Original Penumbra System) but not ADAPT were excluded (19–23). From the remaining 7 articles, 3 were excluded because they used a stent retriever concurrently with the local aspiration technique [stent retriever with local aspiration; CASPER (24), SRLA (10), or Solumbra (25)].

**Figure 1:**
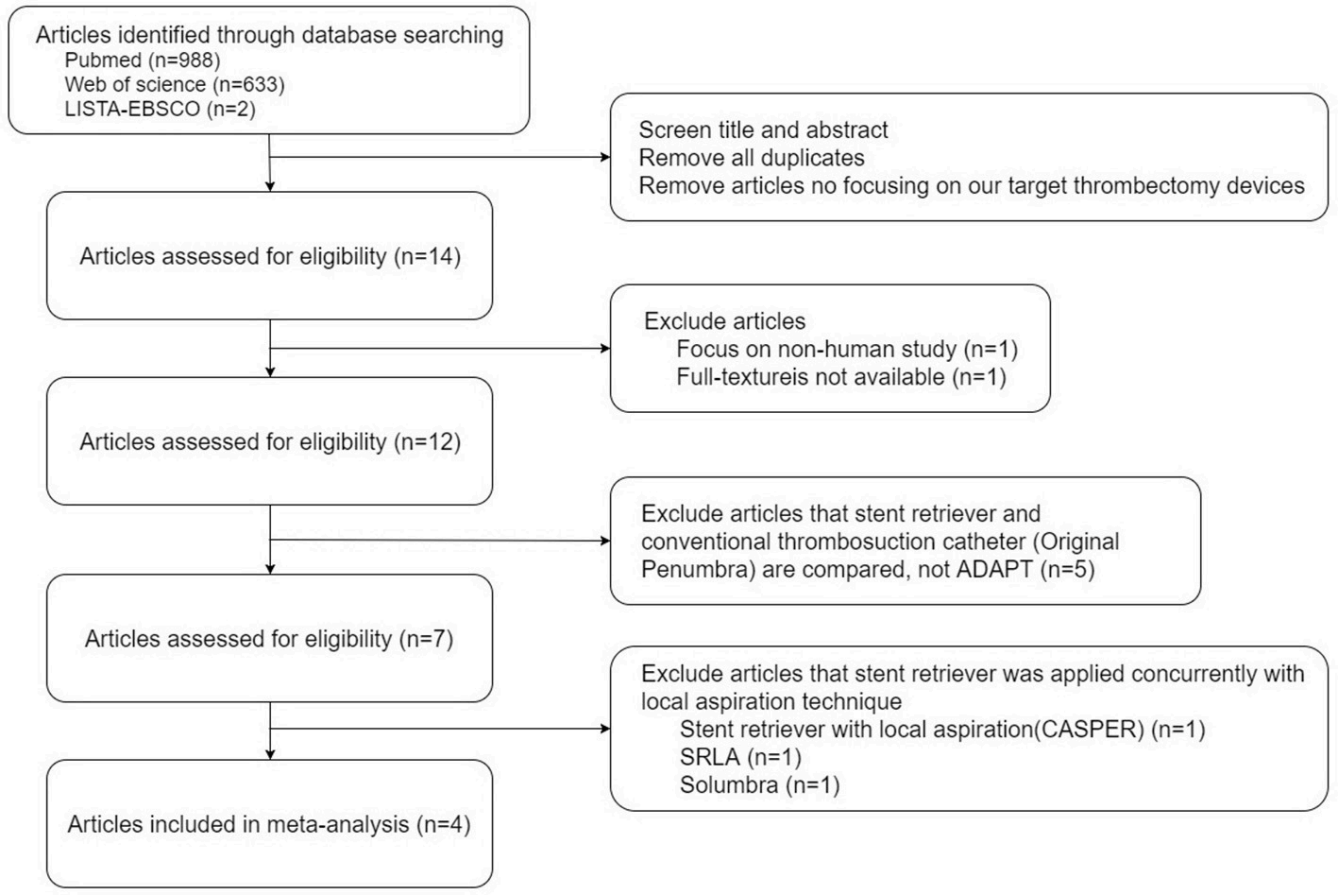
Summary of the evidence search and selection.

Finally, 4 articles were included in our meta-analysis, and the full texts of selected articles were reviewed for further study (17, 26–28) (Figure 1).

### Data extraction and study appraisal

Data were extracted independently by KH. and KC. and verified by other authors. Any disparities in prevalence data were resolved by consensus-based discussions among authors. The following parameters were extracted from the articles: demographic profiles (number of patients, sex, and age), disease status [lesion location and the initial National Institute of Health Stroke Scale (NIHSS)], therapeutic information [the use of intravenous tissue plasminogen activator (IV-tPA)] and the interval between arterial puncture and recanalization), and information regarding applied thrombectomy devices (first-line stent retriever or first-line ADAPT, and the detailed device model). The primary outcome was the proportion of patients with the Thrombolysis in Cerebral Ischemia (TICI) scale of 2b or 3 at the end of all procedures, corresponding to the reperfusion of at least 50% of the affected vascular territory. Secondary outcomes were the proportion of patients with functional independence [the Modified Rankin Scale (mRS) of 0–2 at the third month], the proportion of patients with the Thrombolysis in Cerebral Ischemia (TICI) scale of 2b or 3 by primary chosen device, and the proportion of patients who received rescue therapies. Rescue therapy was defined as the application of other endovascular techniques, devices, or medication different from those used primarily. Safety outcomes were the proportion of patients with symptomatic intracranial hemorrhage (sICH) and the all-cause mortality rate within 3 months.

After the final list of full-text articles was obtained, 2 authors (KH and KC) independently reviewed all the included studies and evaluated them using the Critical Appraisal Skills Programme (CASP) tools, including the CASP randomized controlled trial (RCT) checklist (29) and the CASP cohort study checklist (30), which consisted of 11 questions for RCTs and 12 questions for cohort studies, respectively. The impact of publication bias on the results of the meta-analysis was assessed by using Deek funnel plots (Supplementary Material) (31).

### Statistical analysis

A meta-analysis was conducted using the software Comprehensive Meta-Analysis (Version 3, Biostat, Englewood, NJ, USA). Six major factors, including the primary outcome (the TICI scale at the end of all procedures), secondary outcomes (the mRS at the third month, the TICI scale by primary chosen device, and the proportion of patients who received rescue therapies), and safety outcomes (the proportion of patients with postprocedural sICH and the mortality rate within 3 months), were compared because these factors are of important value in clinical practice and correlate with the long-term outcomes of patients with stroke. Study-level odds ratios (ORs) with 95% confidence intervals (CIs), *P*-values, and results of tests for heterogeneity (Cochran Q test, Tau^2^ value, I^2^ value, and *P*-value) were all calculated. We also assumed that statistical heterogeneity existed when the I^2^ value was higher than 50%. Otherwise, forest plots based on ORs and 95% CIs were illustrated to compare pooled treatment effects and major complications between first-line stent retriever and first-line ADAPT groups.

## Results

### Study characteristics

All 4 studies included in our meta-analysis had been published from 2016 to 2017. Of these 4 studies, one was a RCT, one was a prospective nonrandomized cohort study, and the remaining 2 were retrospective cohort studies. The CASP score of the RCT was 11, and the CASP scores of other non-RCT studies were all greater than or equal to 10. The quality of the evidence of all these studies was satisfactory (Table 1).

**Table 1:** Comparison of major demographic profiles; disease statuses; therapeutic information; the applied thrombectomy devices; and primary, secondary, and safety outcomes of the studies included.

**Author and Year**	**Study design**	**Total number**	**First-line thrombectomy device**	**Rescue therapy (%)**	**Number of patients**	**Sex (M/F)**	**Age (year)**	**NIHSS**	**TICI 2b/3 (%) by primary device**	**TICI 2b/3 (%) at the end of procedures**	**Time to recanalization (minutes)**	**Favorable outcome (mRS score of 0-2) at the third month (%)**	**IV-tPA (%)**	**Lesion location**	**Mortality within 3 months (%)**	**sICH (%)**	**Conclusion**	**CASP score^a^**
Lapergue et al. (17)	RCT	381	Stent retriever (Solitaire FR /Trevo^b^)	45 (23.8)	189	104/85	68.1 ± 14.6	16.1 ± 6.5	128 (67.7)	163 (86.2)	45	91/182 (50.0)	124 (65.6)	ICA-M2	35/182 (19.2)	N/A	Compared with the stent retriever, first-line thrombectomy with contact aspiration did not result in an increased successful revascularization rate at the end of the procedure.	11/11
			ADAPT (5MAX ACE or ACE64^b^)	63 (32.8)	192	103/89	71.7 ± 13.8	16.3 ± 5.9	121 (63.0)	163 (84.9)	38	82/181 (45.3)	126 (65.6)	ICA-M2	35/181 (19.3)	N/A		
Lapergue et al. (26)	Prospective cohort study	243	Stent retriever (Solitaire FR)	7 (5.9)^*^	119	55/64	65.5 ± 14.7	15.9 ± 6.1	N/A	82 (68.9)^*^	50	63 (54.8)	54 (45.4)^*^	ICA MCA	20 (17.4)	7 (5.9)	First-line ADAPT achieved higher recanalization rates than did the Solitaire device.	10/12
			ADAPT (5MAX ACE)	52 (42.0)^*^	124	61/63	64.3 ± 15.7	15.9 ± 6.5	N/A	102 (82.3)^*^	45	61 (53.0)	82 (66.1)^*^	ICA MCA	26 (22.6)	3 (2.4)		
Kim et al. (27)	Retrospective cohort study	41	Stent retriever (Solitaire AB/FR)	2 (12.5)	16	7/9	76.5	10.5	13 (81.2)	14 (87.5)	38.5^*^	12 (75.0)	7 (43.8)	M2	N/A	0 (0)	Stent retriever thrombectomy may provide faster reperfusion than ADAPT, whereas ADAPT might be associated with lower distal embolization and a higher reperfusion rate for the first thrombectomy attempt.	10/12
			ADAPT (041 or 4Max with manual thrombosuction)	5 (20)	25	17/8	71	15	16 (64.0)	18 (72.0)	53^*^	21 (84.0)	13 (52)	M2	N/A	1 (4.0)		
Son et al. (28)	Retrospective cohort study	31	Stent retriever (Solitaire AB)	No	13	7/6	68.9 ± 10.4	27.3 ± 11.0	11 (84.6)	11 (84.6)	N/A	5 (38.5)	5 (38.5)	BA	N/A	2 (15.4)	Thrombosuction devices and Solitaire thrombectomy devices were associated with similar recanalization rates and clinical outcomes in patients with acute basilar artery occlusion. Thrombossuction appeared to allow more rapid and complete recanalization than Solitaire thrombectomy.	10/12
			ADAPT (5MAX with manual thrombosuction)	NO	18	14/4	66.4 ± 11.4	21.3 ± 9.7	18 (100)	18 (100)	N/A	8 (44.4)	9 (50)	BA	N/A	0 (0)		

a *CASP Randomized Controlled Trial Checklist for the first trial and CASP Cohort Study Checklist for the remaining 3 studies*.

b The most common 2 devices used in the treatment group.

* *Significant different between 2 groups*.

*ADAPT, A direct aspiration first-pass technique; TICI, Treatment in Cerebral Infarction; NIHSS, National Institutes of Health Stroke Scale; RCT, Randomized controlled trial; ICA, Internal carotid artery; MCA, Middle cerebral artery; M2, The M2 segment of the middle cerebral artery; BA, Basilar artery; mRS, modified Rankin Scale; IV-tPA, intravenous tissue-type plasminogen activator; sICH, symptomatic intracranial hemorrhage; CASP, Critical Appraisal Skills Programme; N/A, Not available*.

### Patient profiles

In the meta-analysis, 696 patients were eligible for analysis, including 368 men, and 328 women with mean age ranging from 64.3 to 76.5 years. A stent retriever was used as the first-line thrombectomy device in 337 patients (48.4%), and ADAPT was used as the first-line therapy in 359 patients (51.6%). The initial mean NIHSS ranged from 10.5 to 27.3; the mean time to recanalization ranged from 38 to 53 min; the percentage of synchronized IV-tPA usage ranged from 38.5 to 66.1%. The lesions included in these studies were in the internal carotid artery, middle cerebral artery (M1 and M2 segments), and basilar artery (Table 1).

### Primary outcome—TICI scale at the end of all procedures

**Figure 2:**
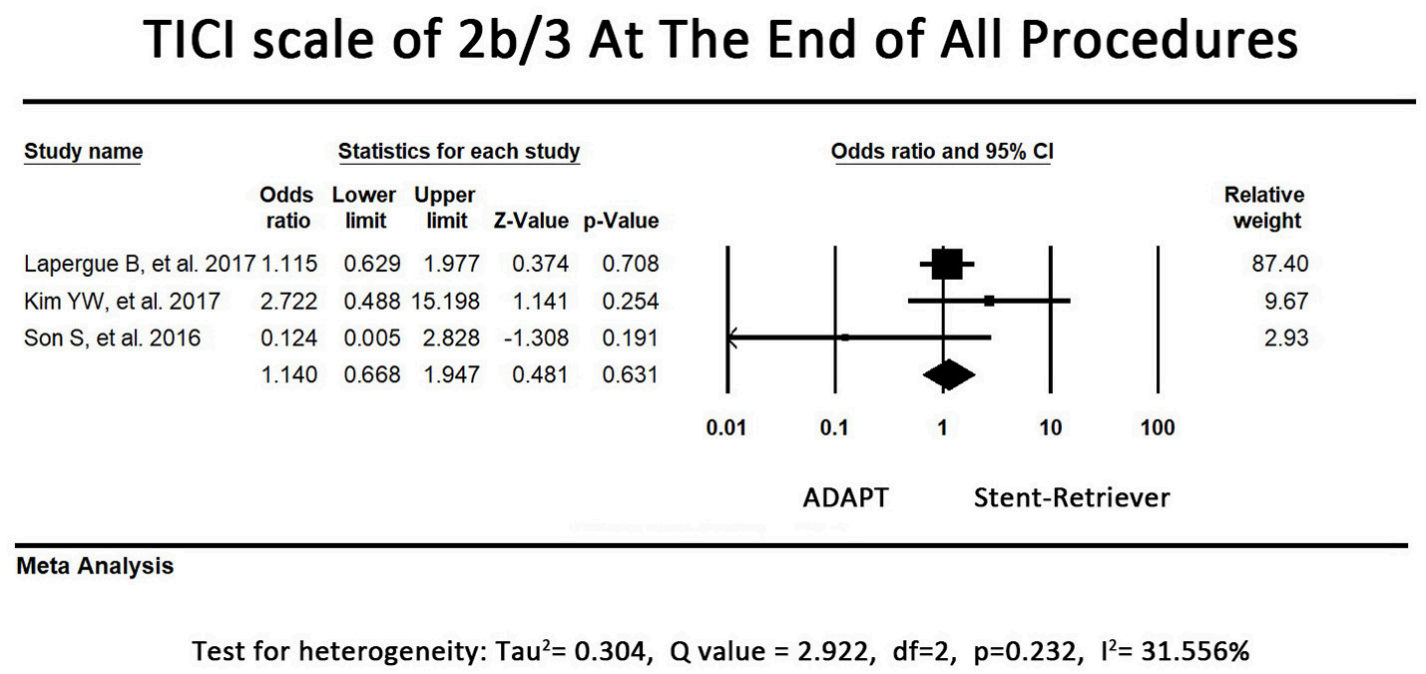
Forest plot of the primary outcome (TICI 2b/3 at the end of all procedures). There is no difference between the first-line ADAPT group and the first-line stent retriever group. (TICI: Treatment in Cerebral Infarction).

Regarding the proportion of patients with TICI scale of 2b or 3 at the end of all procedures, 2 of the included studies reported that the first-line ADAPT group had a higher proportion of patients with TICI scale of 2b or 3. By contrast, the other 2 studies tended to favor first-line stent retriever usage. The pooled primary outcome (TICI scale at the end of all procedures) showed a tendency to be more favorable in the first-line ADAPT group; however, no significant difference was observed between the 2 groups (OR = 0.78, 95% CI = 0.52-1.16, *P* = 0.22). Because there is heterogeneity between studies (I^2^ = 59.43%), we excluded the study conducted by Lapergue et al. (26) to eliminate the heterogeneity. The pooled proportion of patients with TICI scale of 2b or 3 at the end of all procedures were 86.2% in first-line stent retriever group and 84.7% in first-line ADAPT group. The revised primary outcome still showed no significant difference between the 2 groups (OR = 1.14, 95% CI = 0.67-1.95, *P* = 0.63, I^2^ = 31.56%, Figure 2).

### Secondary outcome – mRS at the third month

**Figure 3:**
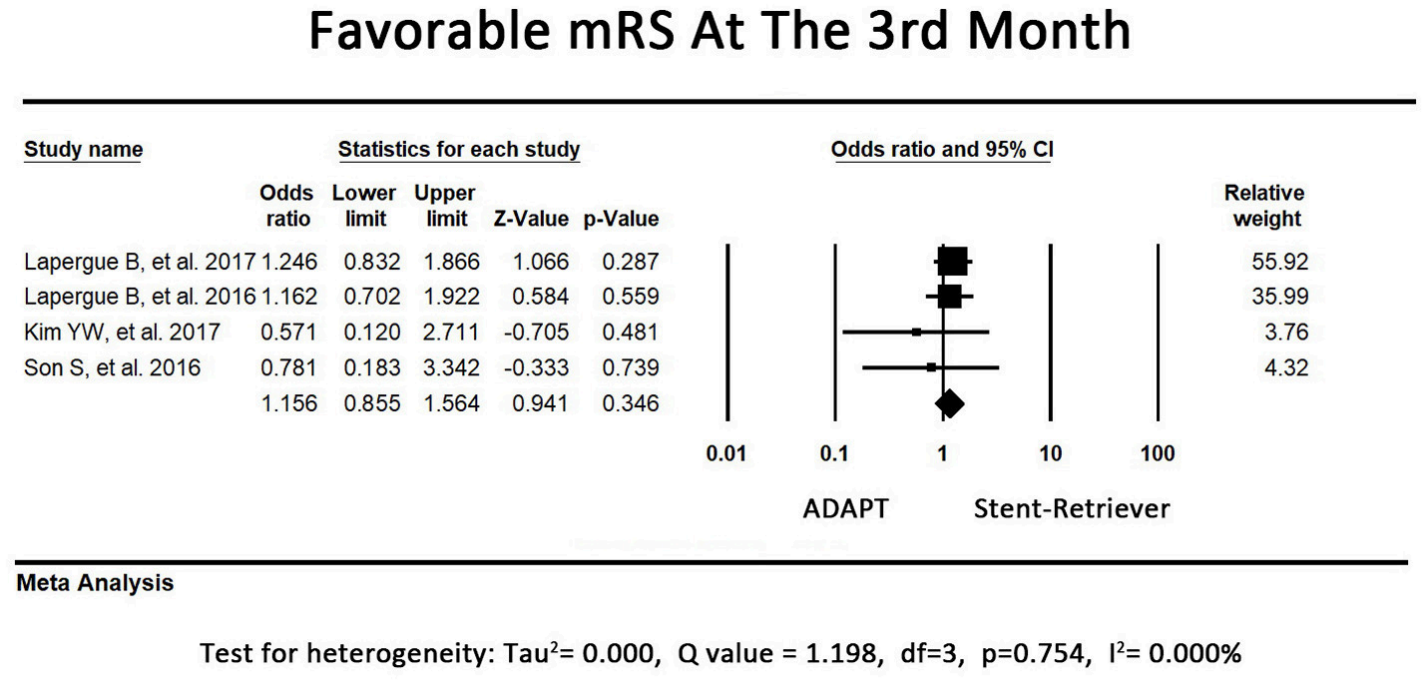
Forest plot for secondary outcome (mRS at the third month). There is no difference between the first-line ADAPT group and the first-line stent retriever group. (mRS: modified rankin scale).

The direction of the effect favored first-line ADAPT use in 2 of the included studies. The other 2 included studies revealed a tendency to be more favorable in the first-line stent retriever group, although adjusted ORs for the treatment were not significant. The pooled rates of functional independence in the third month did not differ between the first-line ADAPT group and the first-line stent retriever group (OR = 1.16, 95% CI = 0.86-1.56, *P* = 0.35, I^2^ = 0.00%, Figure 3).

### Secondary outcome—TICI scale by primary chosen device

**Figure 4:**
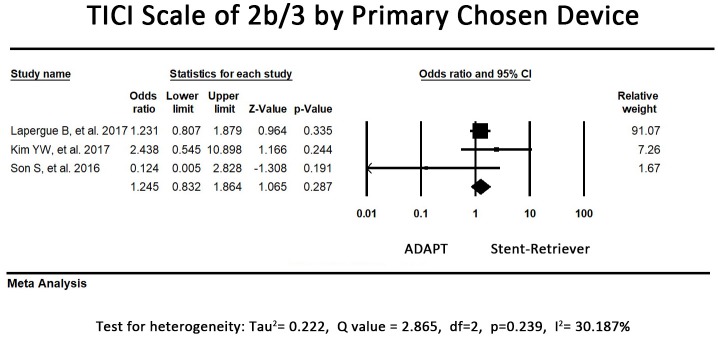
Forest plot for the secondary outcome (TICI 2b/3 by primary chosen device). There is no difference between the first-line ADAPT group and the first-line stent retriever group. (TICI: Treatment in Cerebral Infarction).

Regarding the proportion of patients with TICI scale of 2b or 3 by primary chosen device alone, one study (26) was excluded because the data of TICI scale by primary chosen device was not available. 2 of the included studies reported that the first-line stent-retriever group had a higher proportion of patients with TICI scale of 2b or 3 when stent-retriever was used alone. However, another study tended to favor primary ADAPT usage. The pooled proportion of patients with TICI scale of 2b or 3 by primary chosen device were 69.7% in first-line stent retriever group and 65.6% in first-line ADAPT group. The pooled secondary outcome (TICI scale by primary chosen device) showed no significant difference between the 2 groups (OR = 1.25, 95% CI = 0.83-1.86, *P* = 0.29, I^2^ = 30.19%, Figure 4).

### Secondary outcome—proportion of patients who received rescue therapies

The rescue therapy data was available in 3 articles and the pooled meta-analysis showed significantly higher rates of application of rescue therapy in the first-line ADAPT group (OR = 0.42, 95% CI = 0.28-0.61, *P* < 0.001). The pooled recanalization rates of applied rescue therapy in the stent-retriever group and the first-line ADAPT group were 76.6% and 64.7%, respectively (*p* = 0.17).

### Safety outcomes—sICH

**Figure 5:**
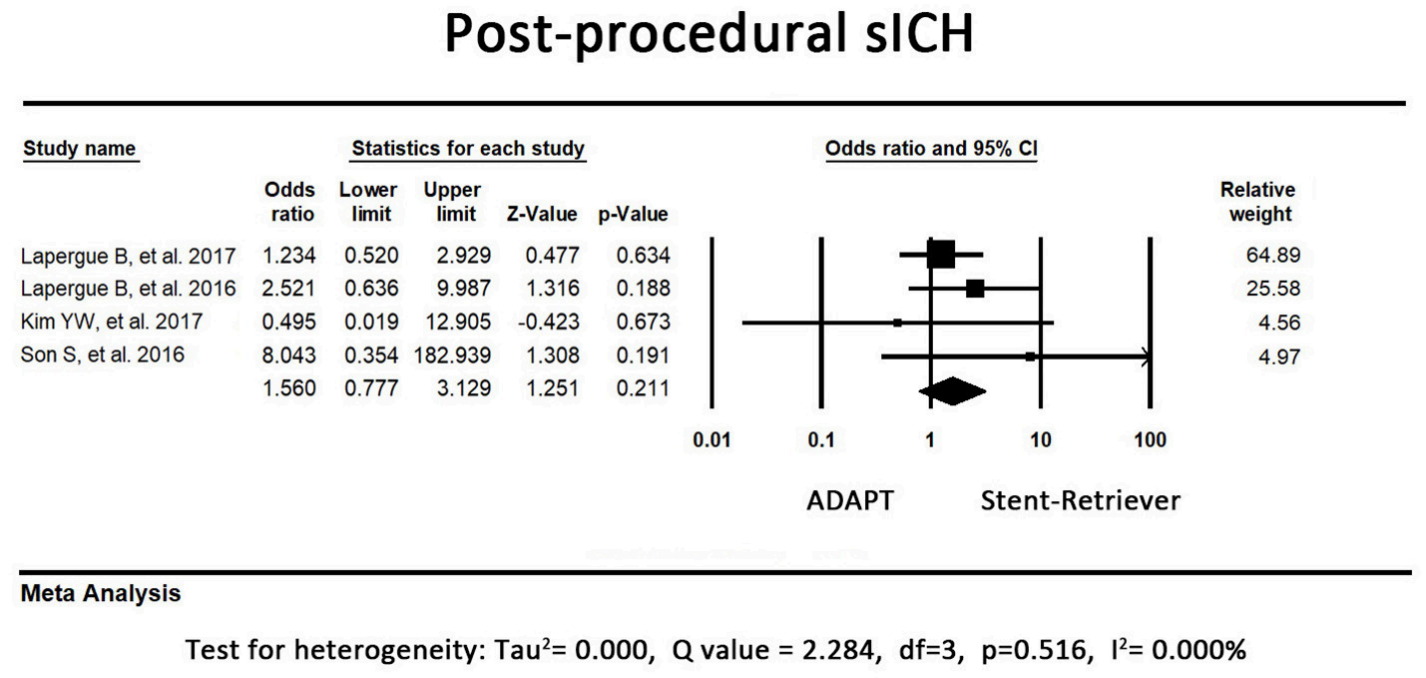
Forest plot for the postprocedural sICH. There is no difference between the first-line ADAPT group and the first-line stent retriever group. (sICH: symptomatic intracranial hemorrhage).

In 3 of the included studies, the first-line stent retriever group tended to have a higher rate of postprocedural sICH. However, an opposite result was observed in the remaining study. The pooled result of sICH did not significantly differ between the first-line ADAPT group and the first-line stent retriever group (OR = 1.56, 95% CI = 0.78-3.13, *P* = 0.21, I^2^ = 0.00%, Figure 5).

### Safety outcomes—all-cause mortality rate within 3 months

**Figure 6:**
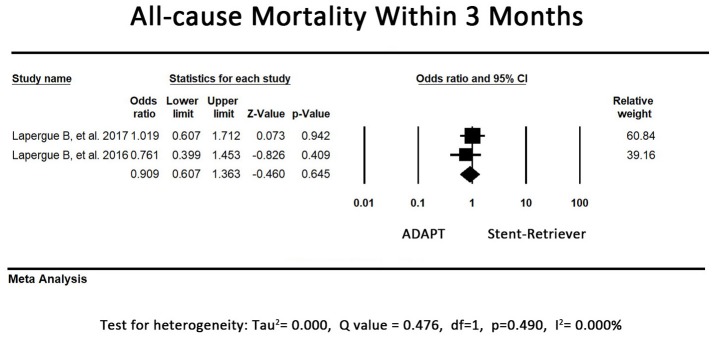
Forest plot for the all-cause mortality rate within 3 months. There is no difference between the first-line ADAPT group and the first-line stent retriever group.

In one study, the first-line stent retriever group tended to have a higher mortality rate within 3 months. Another study revealed a controversial result. The remaining 2 studies did not record the exact time of mortality after the procedure; thus, their results were not included in our analysis. The pooled all-cause mortality rate within 3 months did not differ between the first-line ADAPT group and the first-line stent retriever group (OR = 0.91, 95% CI = 0.61-1.36, *P* = 0.65, I^2^ = 0.0%, Figure 6).

### Comparison of time to recanalization

The time to recanalization was defined as the time interval between arterial puncture and the recanalization of the occluded artery. The weighted average time to recanalization was 46.5 min in the first-line stent retriever group versus 41.6 min in the ADAPT group.

## Discussion

Our results revealed no significant difference between these 2 strategies of management in the primary outcome (TICI scale score at the end of all procedures), secondary outcomes (mRS score in the third month, TICI scale score by primary chosen device), and all safety outcomes (proportion of patients with sICH and the all-cause mortality rate within 3 months). The proportions of patients with TICI scale of 2b or 3 by primary chosen device were not significant different in studies managed occlusions in the M2 segment of MCA (27), basilar artery (28) and internal carotid artery to the M2 segment of MCA (17). The result suggested that the efficacies of stent-retriever and ADAPT were equivalent for clots in these different locations.

In ADAPT, the largest caliber aspiration catheter that the vessel could accommodate was applied, including ACE68, ACE64, ACE60, 5MAX, 4MAX, 041, and 3MAX reperfusion catheters. The coaxial system was navigated and positioned immediately in contact with the clot (32). Aspiration was then performed using either a syringe or the Penumbra aspiration pump. Theoretically, ADAPT should take less time to recanalize the target artery because a stent retriever must be passed through the clot. On the basis of the weighted average data, the procedure time for both techniques was comparable, but ADAPT could be completed marginally faster than the stent retrieval technique (41.6 vs. 46.5 min). However, we could not evaluate whether the procedure time was significantly different between these 2 techniques because most of the studies did not provide detailed statistical data of time to recanalization. It is noteworthy that the overall time spent for revascularization in the 2 groups was equivalent, despite the truth that the ADAPT group applied salvage therapies more frequently. This may be a consequence of the fact that ADAPT does not preclude the operator from incorporating other devices if the front-line aspiration is not working. Having the large bore aspiration catheter at the face of the clot facilitates the use of adjunctive devices for rescue therapies because it provides a direct conduit to the thrombus (9). Since longer time in occlusion has been proved to result in more quantities of tissue at risk of becoming infarcted core (33), ADAPT may provide some advantages in the management of acute stroke.

The reperfusion rate was chosen as the primary outcome because a high reperfusion rate is associated with superior clinical outcomes (1, 2, 4, 5, 7). Obtaining a TICI score of 2b or 3 was defined as successful revascularization. This score was assessed before and after the application of rescue therapies because we would like to assess the efficacy of the first-line endovascular strategy after the entire procedure and when they were used alone. Despite the recanalization rate by primary chosen devices were comparable in both groups (Figure 4), the ADAPT group applied more rescue therapies. It means not all cases failed in first-line device received rescue therapy. The reason why ADAPT group had higher proportion of rescue therapy application may also be related to the advantage of its large bore aspiration system, making it less complicated to apply adjunctive devices such as stent retrievers or angioplasty balloon catheter for rescue therapy. The overall TICI 2b/3 rates at the end of all procedures in both groups were comparable, ranging from 68.9 to 100%. The revascularization rate was higher than that reported for patients treated using IV-tPA, which ranged from approximately 21.25 to 33% (34, 35).

The mRS at the third month was chosen as the major secondary outcome, which is a crucial indicator of functional independence. Our results showed that the functional independence rates were similar in the first-line ADAPT and the first-line stent retriever groups, ranging from 38.5 to 84%. We chose the proportion of patients with postprocedural sICH and the all-cause mortality rate within 3 months as our safety outcomes. Several studies have reported lower rates of symptomatic hemorrhages in ADAPT groups than in stent retriever groups (26, 28). However, our overall results revealed no difference in the proportion of patients with sICH between the 2 groups. The mortality rate within 3 months also showed no difference between the 2 groups, though 2 studies were not included in this analysis because they did not report detailed statistical data regarding mortality (27).

In addition to therapeutic outcomes, the choice of thrombectomy devices is also an issue of value-based care (36). Value in health care is a relationship between cost and clinical outcomes. The most cost-effective method of achieving favorable outcomes in patients with acute stroke with large vessel occlusion has been debated in a new trial (37). On the basis of the practice environment in the United States, the average estimated cost for first-line ADAPT with 5MAX ACE alone was $4,916 per case compared with an estimated cost of $9,620 if a first-line stent retriever was used (38). Thus, it seems that first-line ADAPT is a more cost-effective approach in terms of its technical success rate and functional independence rate compared with the stent retriever technique (10). However, our results also showed a significantly higher rate of application of salvage therapies in the first-line ADAPT group, which indicates that up to 31% of patients who received front-line ADAPT also received additional therapies, mostly stent retrievers, at an additional cost. Therefore, which technique is the most cost effective therapy still remains controversial.

## Limitations

This study is constrained by several limitations. Studies that applied ADAPT and stent retrievers simultaneously as the first-line strategy were excluded because this was not acceptable in our experimental design. The excluded techniques included CASPER (24), SRLA (10), and Solumbra (25). The efficacy of combining ADAPT and stent retrievers to obtain a better rate of recanalization is a potential area of our future work. Second, though we have already excluded studies using different techniques, there is still minor heterogeneity of the ADAPT technique (various catheter sizes and aspiration techniques) and the patient population (includes anterior and posterior circulation stroke) between the included studies. Third, most included ADAPT studies were not RCT and had small sample sizes, which may result in overestimate the effect size of outcomes and limit the interpretation of pooled data. Fourth, the rescue therapies applied in studies are not concordant. ADAPT, stent retrievers, combined techniques and angioplasty with or without stenting were applied as rescue therapies in the study conducted by Lapergue et al. (17). In Kim et al. study, the applied rescue therapies were ADAPT, stent retrievers, and intra-arterial administration of tirofiban (27). In another study conducted by Lapergue et al. (26), ADAPT and stent retrievers were the only applied rescue techniques. Because detailed information of applied rescue therapies in different subgroups was lacking, we could not investigate the effects of different rescue strategies. Fifth, more detailed data for lesions in different locations are not accessible, so we were not able to evaluate the efficacy and safety of these two strategies in different segment and laterality of arteries. Finally, other important factors including the application of a balloon guide catheter and the type of anesthesia has been proved to affect functional outcomes in intra-arterial thrombectomy (39, 40). A comparison between these factors was not performed in this study and will be one of our future investigations.

## Conclusion

Among first-line thrombectomy devices for patients with ischemic stroke, ADAPT with the latest Penumbra thrombosuction system was as efficient and safe as stent retrievers, irrespective of the post-thrombectomy TICI scale, mRS at the third month, sICH and all-cause mortality rate within 3 months.

## Data availability statements

All datasets for this study are included in the manuscript and the supplementary files.

## Author contributions

KL-CH, K-IC, S-JC, and YC involved in article review, data collection, preparation, and revision of the manuscript. H-HW involved in statistical revision. C-YC involved in revision of the manuscript. All authors read and approved the final manuscript.

### Conflict of interest statement

The authors declare that the research was conducted in the absence of any commercial or financial relationships that could be construed as a potential conflict of interest.

## Supplementary material

Data Sheet 1 Funnel plot of standard error by log odds ratio.

Data Sheet 2 PRISMA 2009 checklist.
