# Supplementary figures and images for: First-Line A Direct Aspiration First-Pass Technique vs. First-Line Stent Retriever for Acute Ischemic Stroke Therapy: A Meta-Analysis
Source: Front Neurol. 2018 Sep 25;9:801. doi: 10.3389/fneur.2018.00801 (PMC6167481; doi:10.3389/fneur.2018.00801)

**Funnel Plot of Standard Error by Log odds ratio**

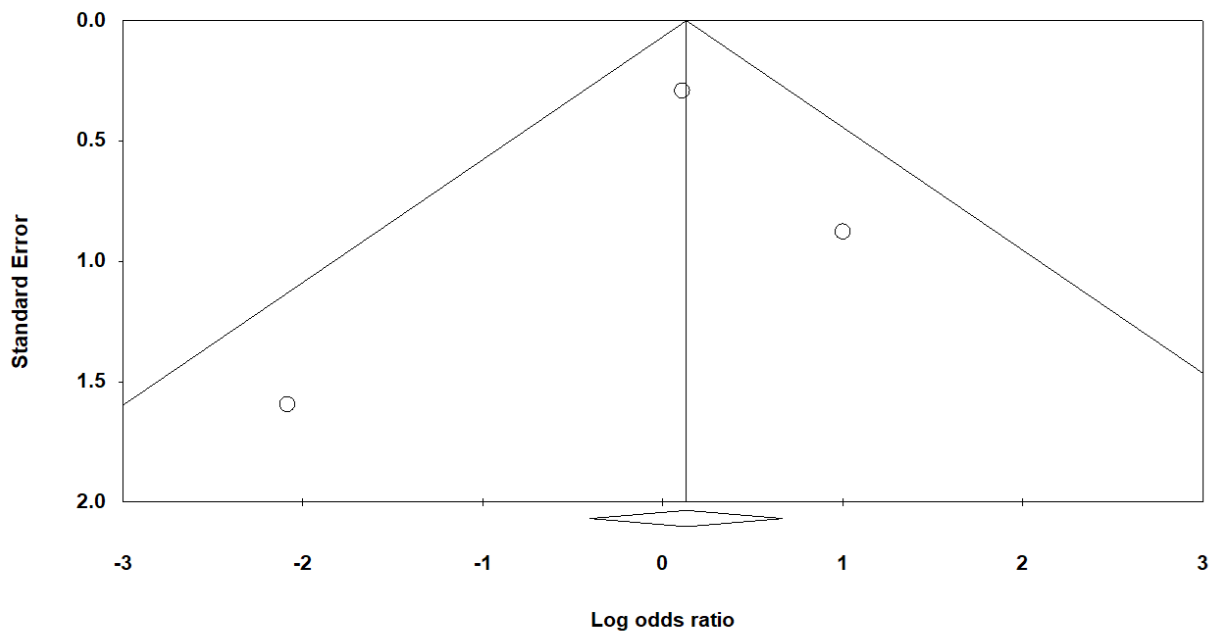

Supplement: Data Sheet 1 — Funnel plot of standard error by log odds ratio. [file Data_Sheet_1.PDF]
